# Green route to synthesis of valuable chemical 6-hydroxynicotine from nicotine in tobacco wastes using genetically engineered *Agrobacterium tumefaciens* S33

**DOI:** 10.1186/s13068-017-0976-9

**Published:** 2017-12-04

**Authors:** Wenjun Yu, Rongshui Wang, Huili Li, Jiyu Liang, Yuanyuan Wang, Haiyan Huang, Huijun Xie, Shuning Wang

**Affiliations:** 1 State Key Laboratory of Microbial Technology, School of Life Science, Shandong University, Jinan, 250100 People’s Republic of China; 2 Institute of Basic Medicine, Shandong Academy of Medical Science, Jinan, 250062 People’s Republic of China; 3 Environment Research Institute, Shandong University, Jinan, 250100 People’s Republic of China

**Keywords:** Nicotine, 6-Hydroxynicotine, 6-Hydroxynicotine oxidase, Biotransformation, Functionalized pyridine, Tobacco wastes, *Agrobacterium tumefaciens*

## Abstract

**Background:**

Tobacco is widely planted as an important nonfood economic crop throughout the world, and large amounts of tobacco wastes are generated during the tobacco manufacturing process. Tobacco and its wastes contain high nicotine content. This issue has become a major concern for health and environments due to its toxicity and complex physiological effects. The microbial transformation of nicotine into valuable functionalized pyridine compounds is a promising way to utilize tobacco and its wastes as a potential biomass resource. *Agrobacterium tumefaciens* S33 is able to degrade nicotine via a novel hybrid of the pyridine and pyrrolidine pathways, in which several intermediates, such as 6-hydroxynicotine, can be used as renewable precursors to synthesize drugs and insecticides. This provides an opportunity to produce valuable chemical 6-hydroxynicotine from nicotine via biocatalysis using strain S33.

**Results:**

To accumulate the intermediate 6-hydroxynicotine, we firstly identified the key enzyme decomposing 6-hydroxynicotine, named 6-hydroxynicotine oxidase, and then disrupted its encoding gene in *A. tumefaciens* S33. With the whole cells of the mutant as a biocatalyst, we tested the possibility to produce 6-hydroxynicotine from the nicotine of tobacco and its wastes and optimized the reaction conditions. At 30 °C and pH 7.0, nicotine could be efficiently transformed into 6-hydroxynicotine by the whole cells cultivated with glucose/ammonium/6-hydroxy-3-succinoylpyridine medium. The molar conversion and the specific catalytic rate reached approximately 98% and 1.01 g 6-hydroxynicotine h^−1^ g^−1^ dry cells, respectively. The product could be purified easily by dichloromethane extraction with a recovery of 76.8%, and was further confirmed by UV spectroscopy, mass spectroscopy, and NMR analysis.

**Conclusions:**

We successfully developed a novel biocatalytic route to 6-hydroxynicotine from nicotine by blocking the nicotine catabolic pathway via gene disruption, which provides an alternative green strategy to utilize tobacco and its wastes as a biomass resource by converting nicotine into valuable hydroxylated-pyridine compounds.

**Electronic supplementary material:**

The online version of this article (10.1186/s13068-017-0976-9) contains supplementary material, which is available to authorized users.

## Background

**Fig. 1:**
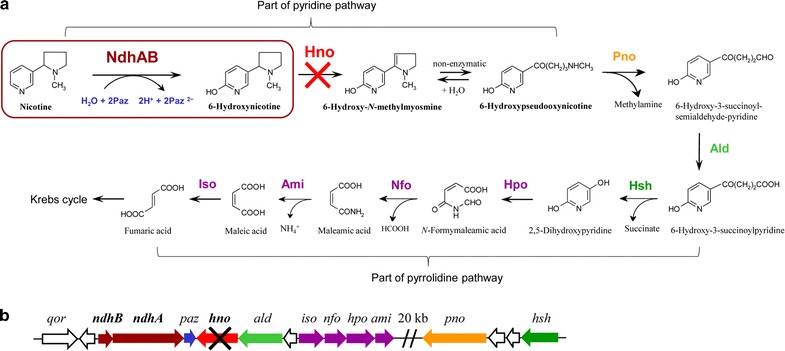
The hybrid pathway for nicotine degradation in *A. tumefaciens* S33 (**a**) and its nicotine-degrading gene cluster (**b**). NdhAB, nicotine dehydrogenase; Paz, pseudoazurin; Hno, 6-hydroxynicotine oxidase; Pno, 6-hydroxypseudooxynicotine oxidase; Ald, putative aldehyde dehydrogenase; Hsh, 6-hydroxy-3-succinoylpyridine hydroxylase; Hpo, 2,5-dihydroxypyridine dioxygenase; Nfo, *N*-formylmaleamate deformylase; Ami, maleamate amidohydrolase (amidase); Iso, maleate *cis*/*trans*-isomerase. The red cross in panel **a** indicates that the pathway has been blocked by disabling the enzyme Hno; the black cross in panel **b** shows our strategy of disrupting the *hno* gene

Tobacco is widely planted as an important nonfood economic crop throughout the world, and large amounts of tobacco wastes are generated during the tobacco manufacturing process [1–3]. Nicotine, the main toxic alkaloid in tobacco leaves and its wastes, comprises approximately 0.6–5% (w/w) of tobacco dry materials and is responsible for tobacco addiction and environmental threats [1, 4]. Nicotine was listed in the Toxics Release Inventory by the US Environmental Protection Agency as early as 1994. It has complex physiological effects and may cause diseases such as cancer [5–7]. Thus, the treatment of nicotine becomes the major concern for tobacco waste disposal. In 2003, the WHO framework convention on tobacco control led to great negative effects regarding the cultivation of tobacco. Therefore, it is imperative to develop efficient technologies to deal with tobacco wastes and utilize tobacco leaves. Nowadays, wastes are often considered as potential resources. Replacing waste disposal with resource recovery is a green method for saving energy [8]. Microbiologists have been engaged in research regarding various applications of tobacco and its wastes because of their high nicotine content, for example, the biotransformation of nicotine into its valuable intermediates, the functionalized pyridines, using nicotine-degrading microorganisms [9–14]. *Agrobacterium tumefaciens* S33, previously isolated from the tobacco rhizosphere, has a strong ability to degrade nicotine via a hybrid of the pyridine and pyrrolidine pathways (Fig. 1a) [4, 11]. It is interesting that at least three intermediates (6-hydroxynicotine, 6-hydroxy-3-succinoylpyridine [HSP], and 2,5-dihydroxypyridine) in this pathway can be used as renewable precursors to synthesize drugs and insecticides via chemical methods. This is due to the hydroxylation of pyridines at the 6-position or 2- and 5-positions, which can be modified easily via specific and efficient biocatalytic processes [10]. 6-Hydroxynicotine and HSP can be used as valuable precursors to synthesize biologically active 2,5- or 3,5-substituted pyridines, such as the insecticide imidacloprid, the anti-Parkinson’s agent SIB-1508Y, and analogs of the potent analgesic Epibatidine, which has obvious paregoric effects [9, 10, 15]. Furthermore, 6-hydroxynicotine and some derivatives perform functions for memory enhancement and oxidation resistance [16], transgene expression induction [17], and microbial resistance [18, 19]. Consequently, using nicotine to produce valuable compounds through a combination of biocatalysis and chemocatalysis offers the possibility for developing new applications for tobacco and its wastes.

Biocatalysis with microbial whole cells is a promising process for industrial production [10, 20]. Compared with chemical methods, biocatalysis is safer, more specific, more sustainable, and more environmentally friendly. The reaction conditions are also less stringent. Compared with the use of enzymes as biocatalysts, whole cells can be repetitively and economically utilized, and their cofactor regeneration is much easier and less expensive [10]. As a result, biocatalysis based on whole cells provides a continuous process for environmentally friendly energy recovery. However, throughout the whole-cell reaction process, the valuable intermediates of nicotine degradation continue to be further catabolized by the wild-type strains cells [9, 12, 19], leading to a low molar conversion and the formation of by-products that cause difficulties for product purification. For this reason, greater efforts are required for the optimization of biocatalyst preparation and catalytic conditions. Recently, great interest has been aroused regarding the development of novel biocatalytic processes to achieve high efficiency and selectivity. Thus, engineered bacteria have been suggested as a potential solution, presenting a new approach for the use and control of microbial transformations [21, 22]. By deleting one or several genes required for catabolism, the nicotine degradation pathway is blocked, and the accumulation of valuable intermediates is achieved [13, 14].

Herein, the efforts were put forth to test the transformation of nicotine into 6-hydroxynicotine by *A. tumefaciens* S33, knowing that this strain harbors nicotine dehydrogenase (NdhAB) that can catalyze nicotine hydroxylation at the 6-position of the pyridine ring with pseudoazurin as its electron acceptor (Fig. 1a) [23, 24]. First, we purified and characterized the key enzyme 6-hydroxynicotine oxidase (Hno) that catalyze the oxidation of 6-hydroxynicotine to 6-hydroxy-*N*-methylmyosmine in *A. tumefaciens* S33. Then, the *hno* gene of strain S33 was disrupted, causing the degradation of nicotine to be blocked at 6-hydroxynicotine (Fig. 1b). After optimizing the reaction conditions and the biocatalyst preparation conditions, we developed a novel and efficient green route for producing 6-hydroxynicotine with the whole cells of the engineered *hno*-disrupted strain S33 as the catalyst.

## Results and discussion

### Purification of Hno from *A. tumefaciens* S33 and identification of its encoding gene

**Fig. 2:**
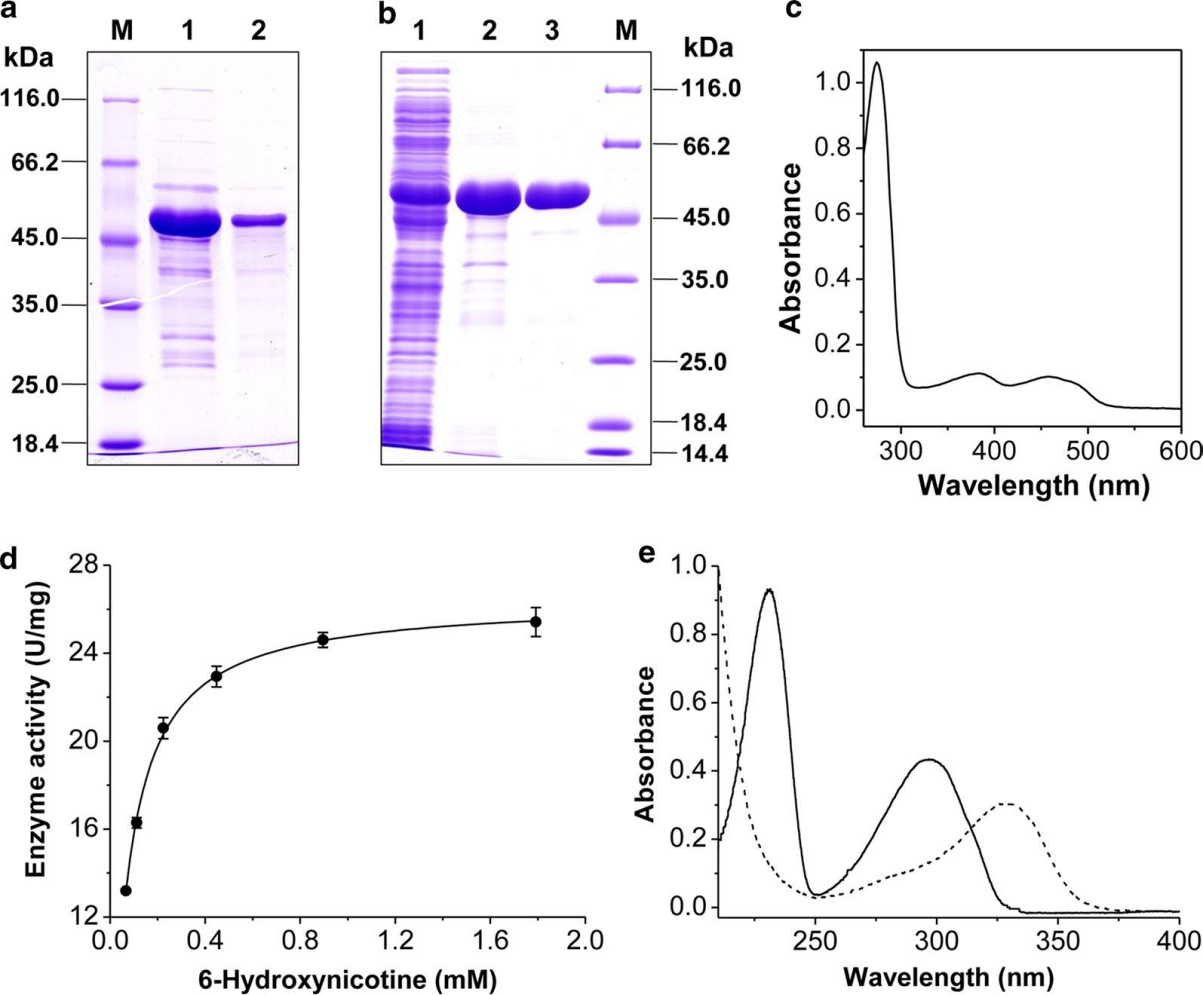
Purification and properties of the Hno from *A. tumefaciens* S33. **a** The wild-type Hno purified from *A. tumefaciens* S33. M, markers; 1, DEAE Sepharose Fast Flow; lane 2, Q Sepharose. **b** The recombinant His-tagged Hno. Lane 1, cell extracts of the recombinant *E. coli* BL21 (DE3); lane 2, His Trap HP; lane 3, DEAE Sepharose Fast Flow; M, markers. **c** UV–visible absorption spectrum of 0.6 mg mL^−1^ purified recombinant His-tagged Hno in 50 mM sodium phosphate buffer (pH 7.0). **d** Determination of the kinetic constants from the 6-hydroxynicotine oxidation. **e** UV–visible absorption spectra of the substrate 6-hydroxynicotine (solid line) and the products of 6-hydroxynicotine oxidation catalyzed by purified recombinant Hno (dashed line)

In our previous study on purification of NdhAB, three yellow-colored fractions adjacent to the NdhAB-containing fractions were found to have 6-hydroxynicotine oxidation activity after separation by DEAE Fast Flow column [24]. The protein was further purified by applying to Q Sepharose and eluted with 0.25 M NaCl, which was nearly pure, as detected by SDS-PAGE (Fig. 2a), and presented a specific 6-hydroxynicotine oxidation activity of 12.0 ± 1.4 μmol min^−1^ mg^−1^. To identify its encoding genes, the purified protein was analyzed using MALDI-TOF/MS. The MS results were searched against the annotated genome of *A. tumefaciens* S33 [25, 26]. We found that the enzyme was encoded by an ORF of 1314 bp, designated as *hno*, which formed one large gene cluster together with the genes for NdhAB, Pno, and Hsh (Fig. 1b); however, its transcription direction was opposite to *ndhAB*. The identification of the *hno* gene was supported also by our previous transcriptomic analysis, where a log_2_ ratio (FPKM of Nic/FPKM of Glu) (FPKM, fragments per kilobase per million) of 5.9 for the *hno* gene was observed in nicotine medium (Nic) and glucose–ammonium medium (Glu) [25]. The deduced protein sequence (437 amino acids, 48.76 kDa) has 99.8% identity to both 6-hydroxynicotine oxidase NctB from *Shinella* sp. HZN7 [27] and 6-hydroxynicotine oxidase VppB from *Ochrobactrum* sp. SJY1 [28], 38.9% to nicotine oxidase Nox from *Pseudomonas* sp. HZN6 [29], 38.4% to nicotine oxidoreductase NicA2 from *P. putida* S16 [30], and 24.8% to 6-hydroxy-l-nicotine oxidase from *A. nicotinovorans* [31, 32]. All these proteins harbor a conserved FAD-binding motif, suggesting that Hno in strain S33 is also a flavin-containing oxidoreductase.

### Heterologous production and properties of recombinant Hno

In order to confirm the function of the *hno* gene, we heterologously expressed it in *E. coli* BL21 (DE3) with a His tag at the N-terminus and purified the recombinant protein using His Trap HP and DEAE Sepharose Fast Flow columns (Fig. 2b). A single band with an apparent molecular mass of 50 kDa was detected by SDS-PAGE, which was identical to the calculated molecular mass of the His-tagged Hno and wild-type Hno purified from *A. tumefaciens* S33 (Fig. 2a). HPLC analysis showed that the yellow protein contained a FAD, which was consistent with the fact that the protein harbors a Rossmann-like domain, as shown by conserved domain analysis in NCBI, and exhibited a typical absorption of flavin at 382 and 459 nm (Fig. 2c). Enzyme assays showed that at 37 °C and pH 9.2 (100 mM Glycine–NaOH buffer), the enzyme showed a *V*_max_ of 26.43 ± 0.16 U/mg, and an apparent *K*_*m*_ for 6-hydroxynicotine of 0.067 ± 0.001 mM (Fig. 2d), which are both similar to the kinetic constants of NctB and VppB from *Shinella* sp. HZN7 [27] and *Ochrobactrum* sp. SJY1 [28], respectively.

**Fig. 3:**
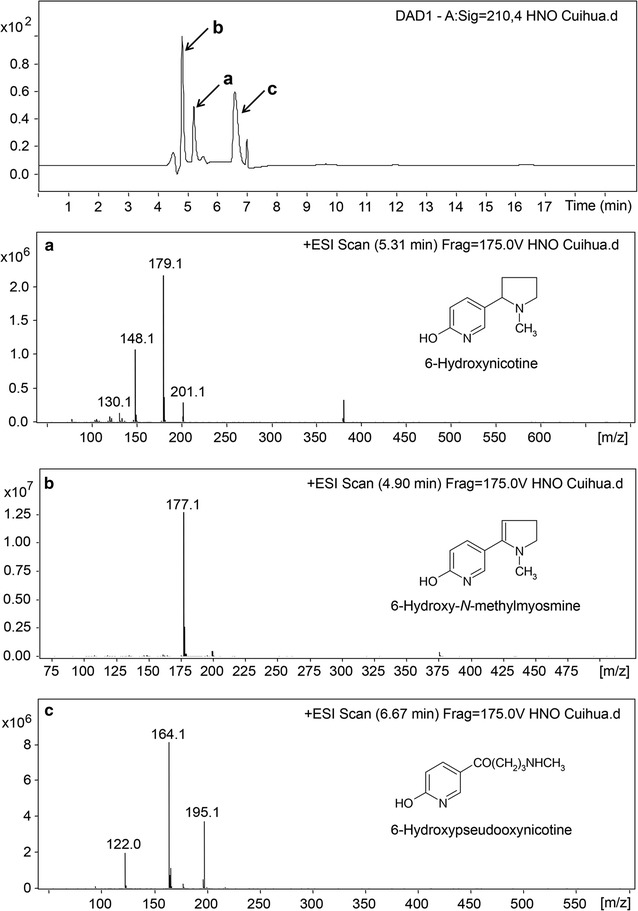
LC–MS profiles of the reaction catalyzed by recombinant Hno from *A. tumefaciens* S33. The HPLC profile was obtained by monitoring with a PDA detector at 210 nm; **a**–**c** the mass spectra of the substrate 6-hydroxynicotine (*m/z* 179.1) and the products 6-hydroxy-*N*-methylmyosmine (*m/z* 177.1) and 6-hydroxypseudooxynicotine (*m/z* 195.1), respectively. Positively charged ions were detected

The reaction products catalyzed by the purified Hno were analyzed by UV–visible spectrophotometer. The absorption spectra (Fig. 2e) showed that the absorption of the substrate 6-hydroxynicotine (maximum absorption at 295 nm) decreased and a new peak appeared (maximum absorption at 334 nm), indicating that 6-hydroxynicotine was transformed into other compounds. The products were further analyzed by LC–MS (Fig. 3). Three large peaks were found in the chromatography (retention time as 4.90, 5.31, and 6.67 min, respectively) (Fig. 3), and monitored with a photodiode array (PDA) detector. The mass-to-charge ratios (*m/z*) of the main fragments in the mass spectra are identical to the calculated molecular mass of 6-hydroxynicotine (peak a, *m/z* 179.1), 6-hydroxy-*N*-methylmyosmine (peak b, *m/z* 177.1), and 6-hydroxypseudooxynicotine (peak c, *m/z* 195.1). This indicates that Hno transforms 6-hydroxynicotine into the 6-hydroxy-*N*-methylmyosmine, which then is converted spontaneously into 6-hydroxypseudooxynicotine, just like the reaction catalyzed by 6-hydroxy-l-nicotine oxidase in the pyridine pathway of *A. nicotinovorans* [33]. These results confirm that Hno from strain S33 is a FAD-containing oxidase and that it catalyzes the second step of 6-hydroxynicotine oxidation in the hybrid nicotine degradation pathway.

### Construction of the strain with disrupted *hno* gene and its complementation strain

**Fig. 4:**
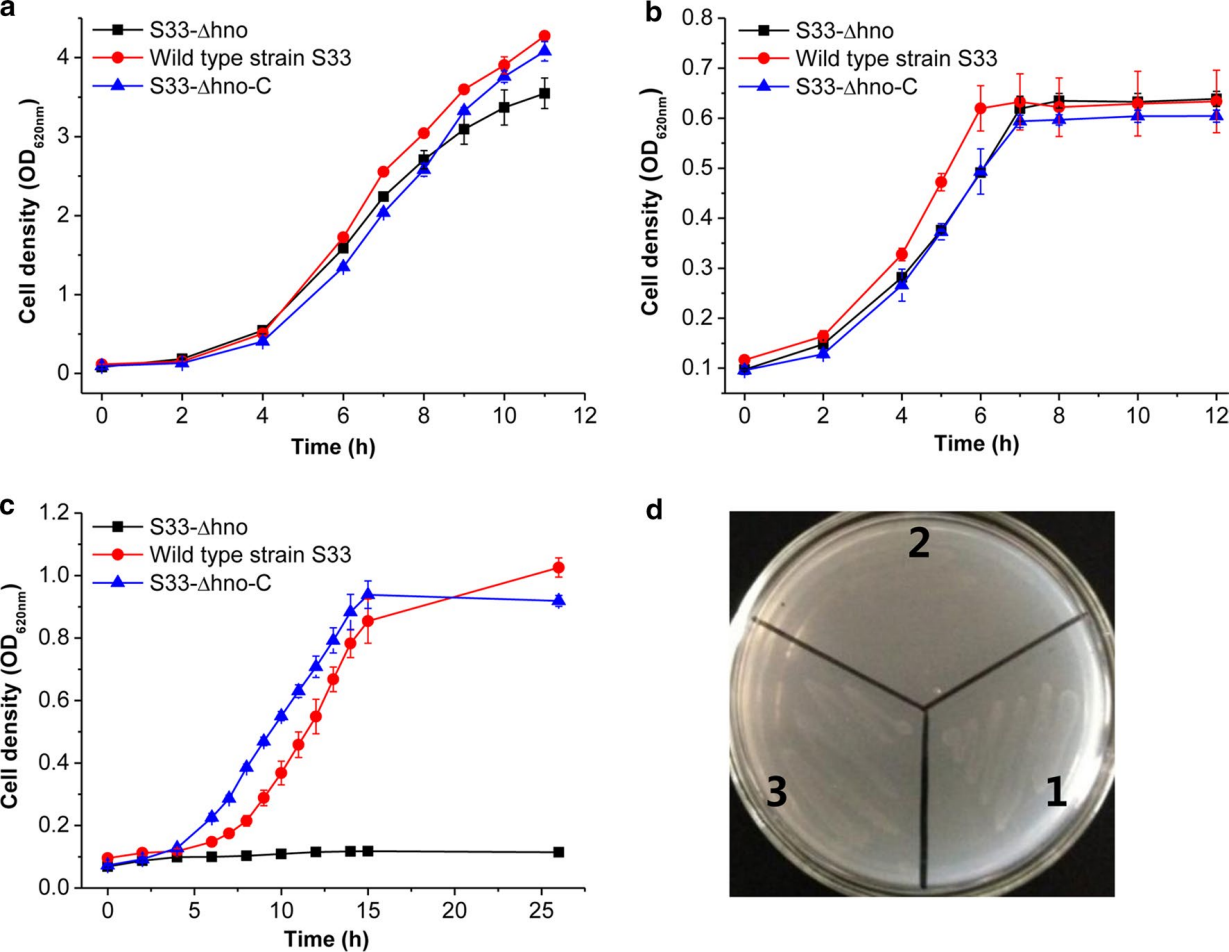
Growth of wild-type *A. tumefaciens* S33, *A. tumefaciens* S33-∆*hno*, and *A. tumefaciens* S33-∆*hno*-C in LB (**a**), HSP (**b**), nicotine (**c**), and 6-hydroxynicotine (**d**) media. In panel **d**: 1, wild-type strain S33; 2, strain S33-∆*hno*; 3, strain S33-∆*hno*-C

To block the 6-hydroxynicotine oxidization by Hno in the nicotine-degrading pathway of strain S33, we disrupted the *hno* gene with the plasmid pJQ200SK harboring the truncated target gene and then complemented with the plasmid pBBR1MCS-5 containing a complete copy of *hno*. Growth tests showed that the mutant strain S33-∆*hno* could grow well in lysogeny broth (LB) (Fig. 4a) and HSP media (Fig. 4b) but not in nicotine (Fig. 4c) or 6-hydroxynicotine media (Fig. 4d), while the wild-type strain and the complemented strain S33-∆*hno*-C could grow in all media. The results confirm that the *hno* gene is responsible for the oxidation of 6-hydroxynicotine and is required for nicotine degradation in strain S33. The mutant strain S33-∆*hno* lost the ability to further catabolize 6-hydroxynicotine.

The activities of NdhAB and Hno in all strains were measured when they were grown in HSP medium or nicotine–glucose–ammonium medium (Table 1). The results showed that Hno activity was not detected in the mutant strain S33-∆*hno*. Interestingly, when strain S33-∆*hno* was cultured in HSP medium, its Ndh activity was much higher than when cultured in nicotine–glucose–ammonium medium.

**Table 1:** The NdhAB and Hno activity of wild-type *A. tumefaciens* S33, *A. tumefaciens* S33-∆*hno*, and *A. tumefaciens* S33-∆*hno*-C cultured in HSP medium (a) or nicotine–glucose–ammonium medium (b)

Enzyme	Sp act (U/mg)-a			Sp act (U/mg)-b		
	Wild-type S33	S33-∆*hno*	S33-∆*hno*-C	Wild-type S33	S33-∆*hno*	S33-∆*hno*-C
NdhAB	0.47 ± 0.019	0.53 ± 0.021	0.52 ± 0.025	0.42 ± 0.018	0.03 ± 0.005	0.14 ± 0.013
Hno	0.05 ± 0.008	< 0.001	0.01 ± 0.002	0.07 ± 0.010	< 0.001	0.03 ± 0.007

### Testing the biotransformation of nicotine into 6-hydroxynicotine catalyzed by whole cells of *A. tumefaciens* S33-∆*hno*

**Fig. 5:**
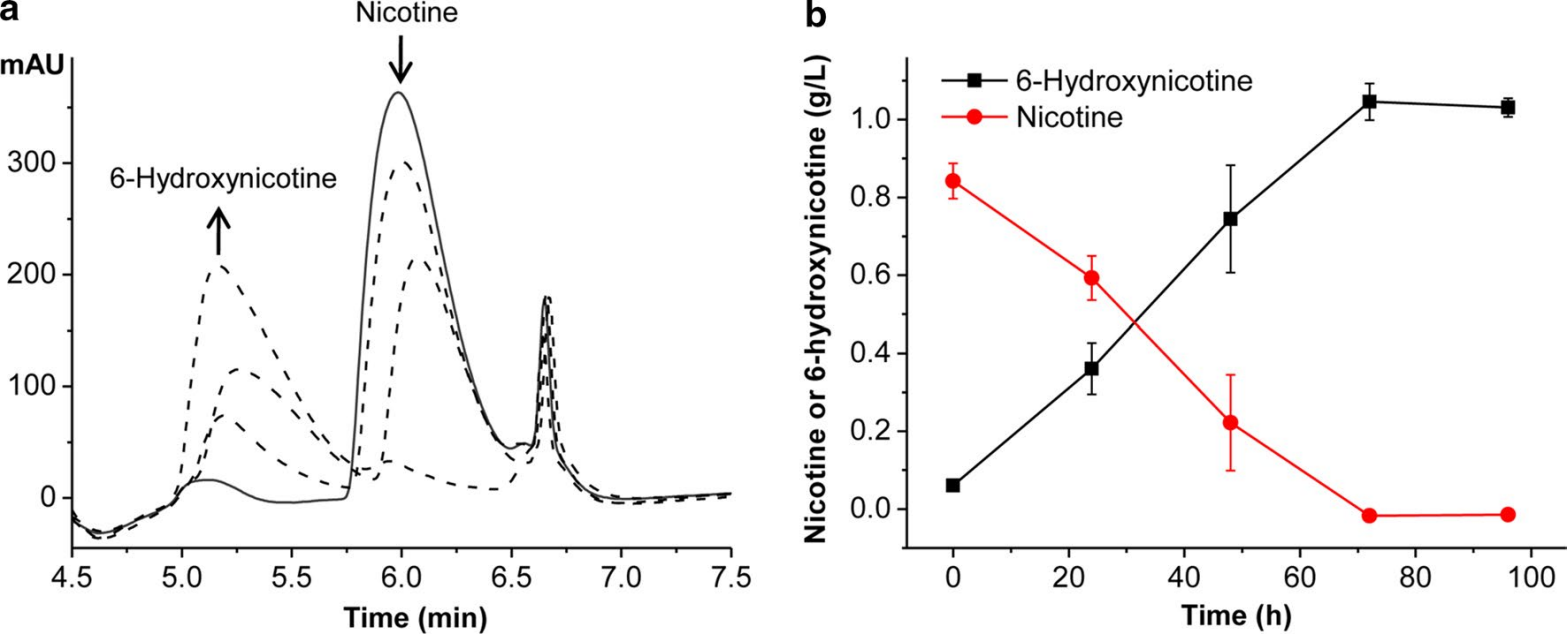
Biotransformation of nicotine into 6-hydroxynicotine catalyzed by the whole cells of *A. tumefaciens* S33-∆*hno*. **a** HPLC profile of the reaction mixtures sampled at different time after starting the reaction. The increase and decrease of the peaks during the reaction are indicated by upward or downward arrows, respectively. **b** Time course of the biotransformation reaction

To determine the possibility of 6-hydroxynicotine production from nicotine, the biotransformation reaction was performed using whole cells of *A. tumefaciens* S33-∆*hno* (~ 1.45 g L^−1^, dry cell weight, DCW; ~ 3.6 OD_620 nm_; one OD unit = 0.41 g L^−1^ DCW) as a catalyst and 0.85 g L^−1^ nicotine as the substrate in 50 mM sodium phosphate buffer (pH 7.0). The cells were cultured with nicotine–glucose–ammonium medium in consideration of the high cost of HSP, where nicotine could induce the expression of the *ndhAB* gene according to the results in Table 1 and previous investigations [24, 25]. The mixture was shaken at 30 °C with a speed of 200 rev min^−1^ (rpm). Samples were withdrawn and analyzed by HPLC to monitor the consumption of the substrate and the formation of the product. Results showed a significant increasing peak with retention time at 5.2 min and a decreasing peak with retention time at 6.0 min (Fig. 5a), which correspond to 6-hydroxynicotine and nicotine, respectively. Finally, nicotine can be converted completely into 6-hydroxynicotine (Fig. 5b). However, the specific catalytic activity of ~ 0.01 g 6-hydroxynicotine h^−1^ g^−1^ dry cells was very low, which needs to be improved.

### Optimization of the reaction conditions for the biotransformation of nicotine into 6-hydroxynicotine

**Fig. 6:**
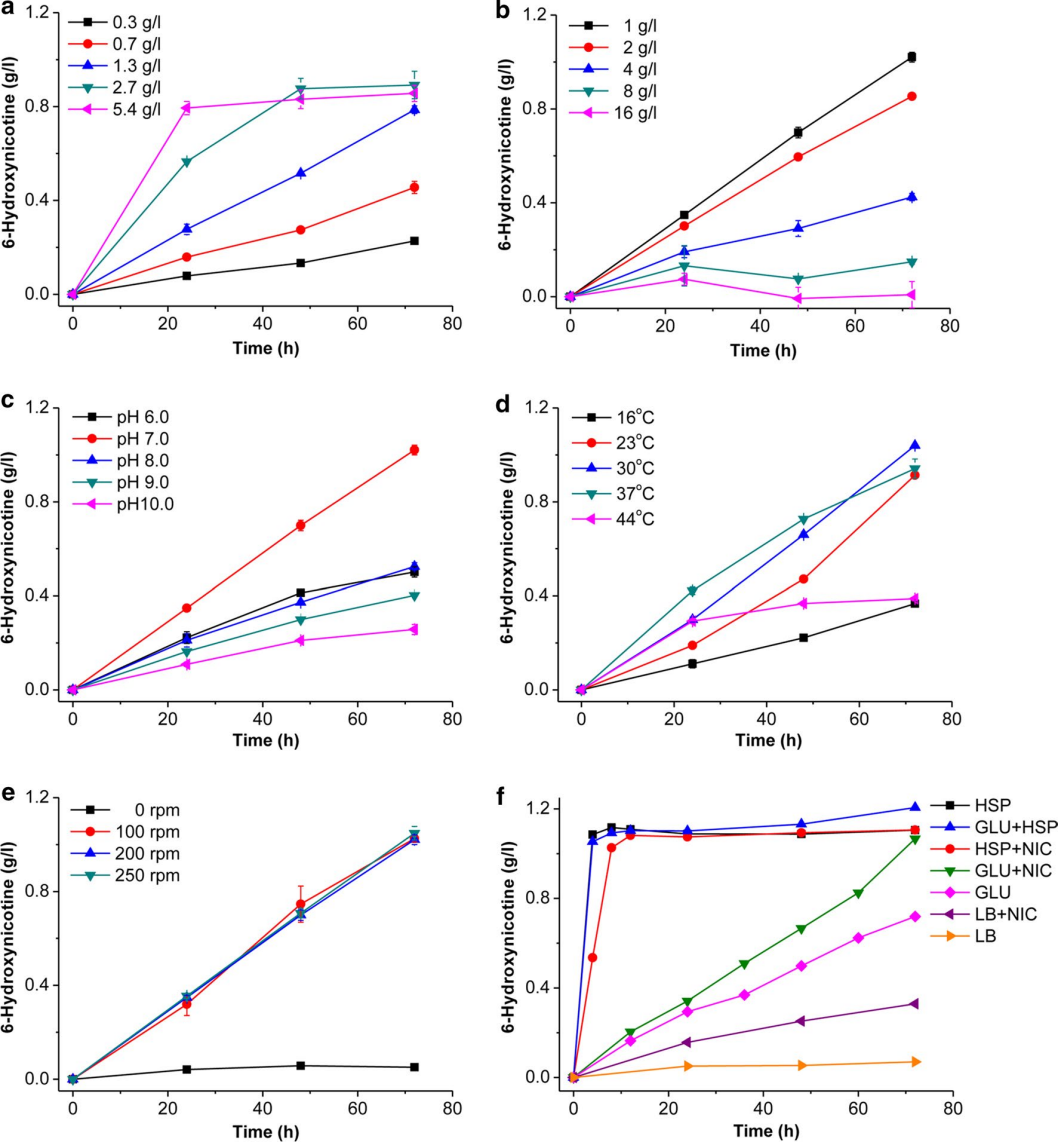
Optimization of biotransformation conditions to produce 6-hydroxynicotine. Effects of the amount of the catalyst added (dry cell weight, DCW) (**a**), the initial concentration of nicotine (**b**), pH (**c**), temperature (**d**), rotation speed (**e**), and the media used for preparation of the biocatalyst (**f**). In panel **c**: 50 mM sodium phosphate buffer (pH 6.0 and pH 7.0), 50 mM Tris–HCl buffer (pH 8.0), and 50 mM glycine–NaOH buffer (pH 9.0 and pH 10.0) were used. In panel **f**: HSP, HSP medium; GLU + HSP, glucose–ammonium–HSP medium; HSP + NIC, HSP-nicotine medium; GLU + NIC, glucose–ammonium–nicotine medium; GLU, glucose–ammonium medium; LB + NIC, LB-nicotine medium; LB, lysogeny broth. The reactions were carried out at 30 °C in 50 mM sodium phosphate buffer (pH 7.0) with 1.0 g L^−1^ nicotine as the substrate and ~ 1.5 g L^−1^ DCW whole cells of *A. tumefaciens* S33-∆*hno*, or as indicated

To improve the catalytic rate, reaction conditions were optimized. As shown in Fig. 6, the catalytic rate was affected by the amount of *A. tumefaciens* S33-∆*hno* whole cells added, the initial concentration of nicotine, pH of the buffer, and the incubation temperature. The catalytic rate rose remarkably with the increasing dry weight of the catalyst (Fig. 6a) and the decreasing initial concentration of nicotine (Fig. 6b); however, the specific catalytic rate did not continue to increase when above 2.7 g L^−1^ of biocatalyst was added (Fig. 6a). The high concentration of nicotine inhibited the catalytic activity. The optimal pH and temperature for the reaction was around 7.0 (Fig. 6c) and 30 °C (Fig. 6d), respectively, which was in consistent with the optimal growth conditions for *A. tumefaciens* S33 [4]. Figure 6e shows that oxygen was essential for the reaction although the key enzyme catalyzing nicotine hydroxylation is a pseudoazurin-dependent NdhAB [23]. We speculate that oxygen is the final electron acceptor of nicotine oxidative degradation, which accepts the electron from the reduced pseudoazurin via the respiratory chain. Thus, the pseudoazurin can be regenerated in the biotransformation reaction. For the tested system, a low rotation rate supplied enough oxygen to the reaction (Fig. 6e). Finally, the preparation of the whole-cell biocatalyst was examined. Different media were used for culturing *A. tumefaciens* S33-∆*hno* (Fig. 6f), where nicotine or HSP was used as an inducer for expressing the nicotine-degrading enzymes, including NdhAB [24, 25]. The results showed that the cells grown in the media containing HSP presented higher catalytic efficiency, which was even better than those grown on nicotine, while LB was the worst medium for preparing the biocatalyst. This agreed with the results from the enzyme assay for the cell extracts of *A. tumefaciens* S33-∆*hno* (Table 1), where HSP demonstrated a better induction for NdhAB activity. In consideration of the cost of HSP and biomass obtained, we chose the glucose–ammonium–HSP medium to culture *A. tumefaciens* S33-∆*hno*.

### Batch and fed-batch biotransformation reactions

**Fig. 7:**
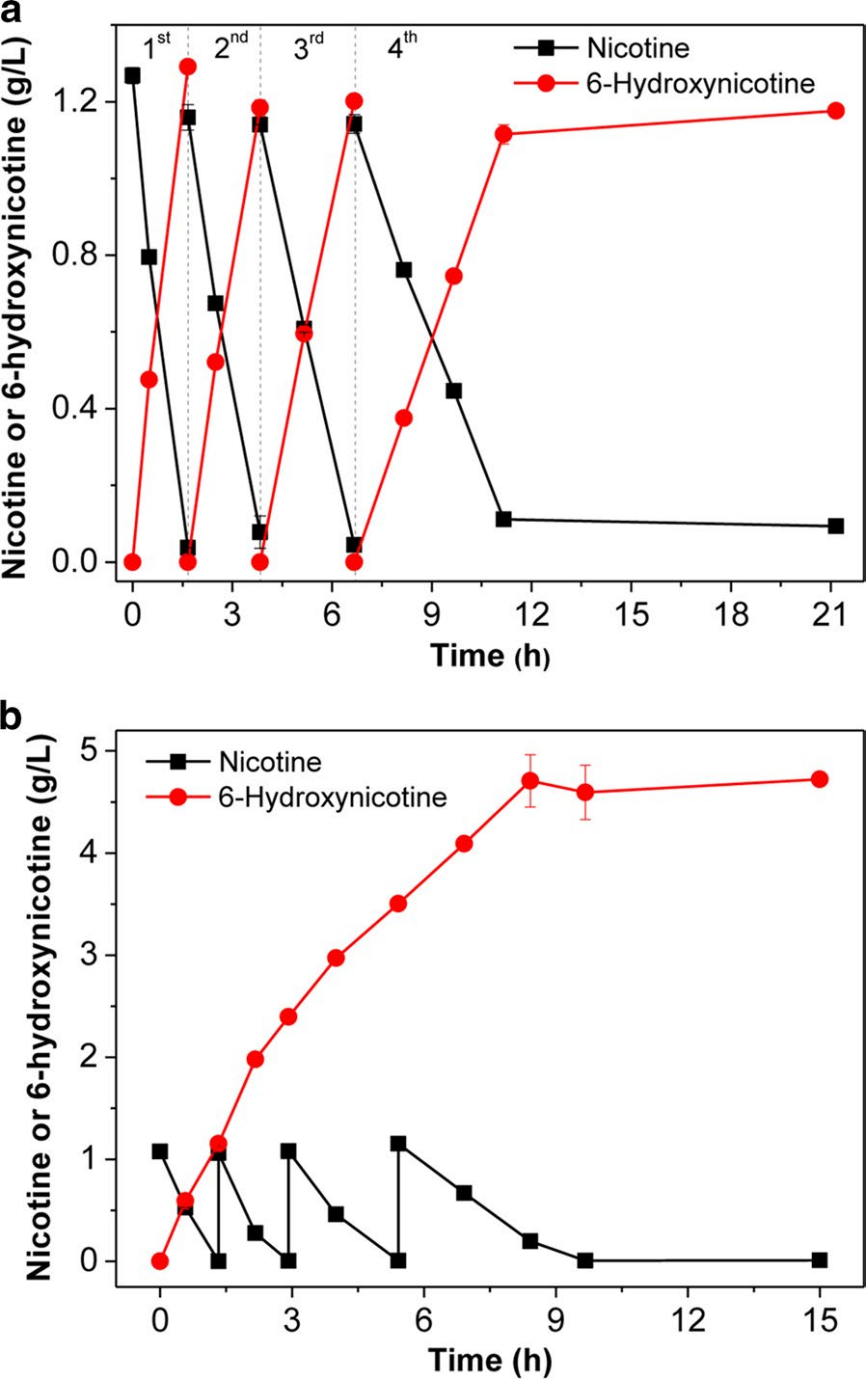
Batch and fed-batch biotransformation reactions converting nicotine into 6-hydroxynicotine under the optimal conditions. **a** Time course of the batch biotransformation reactions. The whole cells were re-used three times after the initial reaction. **b** Time course of the fed-batch biotransformation reaction. Nicotine was supplemented three times

Under the optimal conditions (pH 7.0 and 30 °C), we performed the reaction in 200 mL reaction mixture containing 1 g L^−1^ nicotine and ~ 1.0 g L^−1^ DCW whole cells of *A. tumefaciens* S33-∆*hno* cultured with glucose–ammonium–HSP medium. For batch biotransformations, the catalyst was collected by centrifugation after the first batch reaction and was re-used for three subsequent reactions. As shown in Fig. 7a, the whole-cell biocatalyst could maintain high activity even after being used in four separate reactions. The highest specific catalytic activity reached approximately 1.01 g 6-hydroxynicotine h^−1^ g^−1^ dry cells. For each batch, the molar conversion reached ~ 95%. Due to the inhibitory nature of nicotine (Fig. 6a), we also performed a fed-batch biotransformation (Fig. 7b). During the reaction, we supplemented 1 g L^−1^ of nicotine three times. In total, 4 g L^−1^ of nicotine could be transformed completely into 4.70 g L^−1^ of 6-hydroxynicotine (Fig. 7b). The molar conversion reached ~ 98.4%. However, the catalytic rate gradually decreased as time increased in both batch and fed-batch reactions, which may be due to poor stability of the NdhAB activity in the cells, as previously indicated [23, 24]. These results suggest that a combination of fed-batch reactions and recycled biocatalyst reactions would be feasible and ideal for a large scale biotransformation process. Compared with the growing system developed for producing 6-hydroxynicotine from nicotine by wild-type strain *Arthrobacter oxydans* [9], the catalytic process developed in this study presented both higher conversion rates and higher yields. Moreover, the whole cells biocatalysts could be recovered easily by centrifugation and repeatedly used several times, which is helpful for reducing costs and for product purification.

### Purification and identification of the product 6-hydroxynicotine

**Fig. 8:**
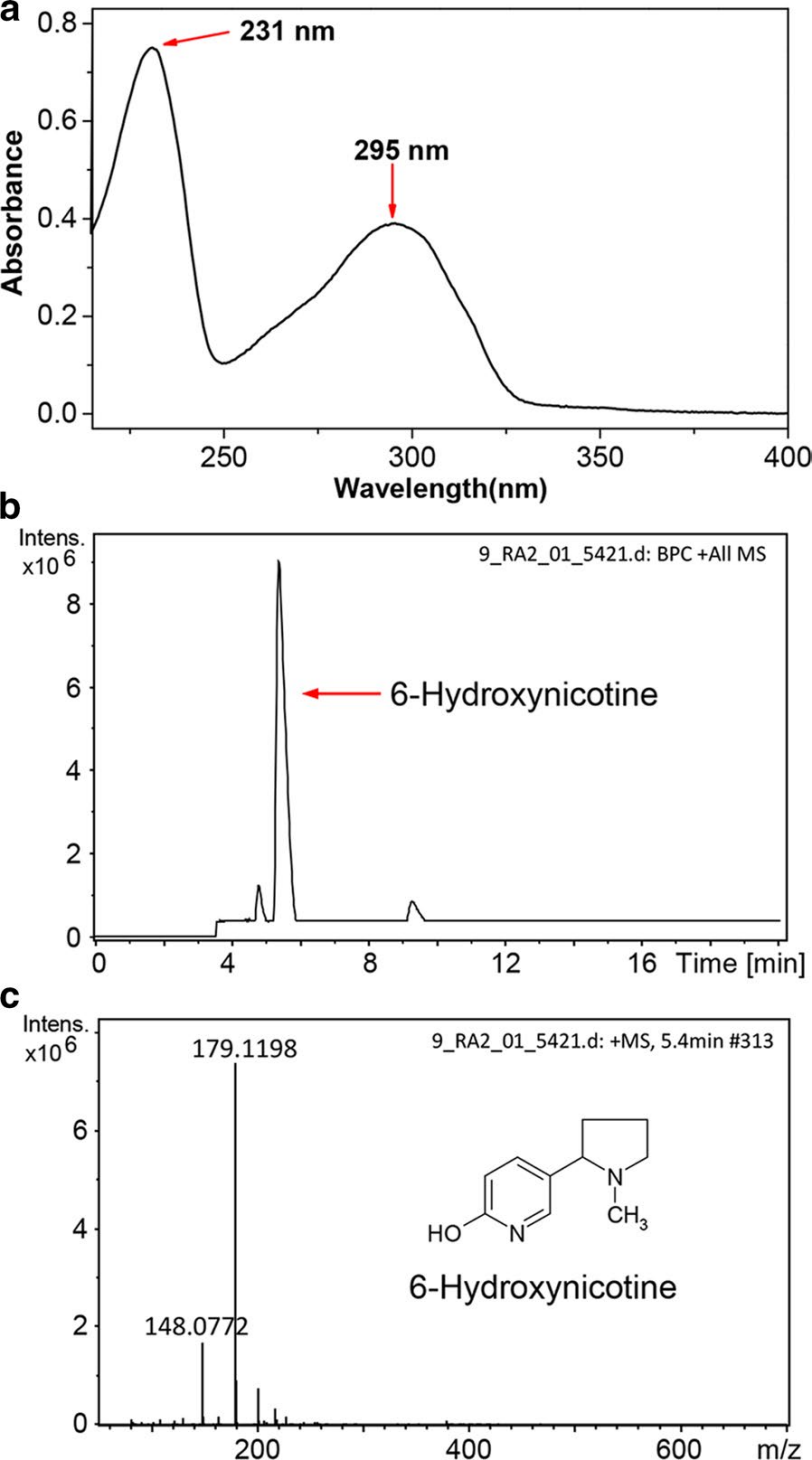
Identification of the product 6-hydroxynicotine purified from the biotransformation reaction mixture. **a** UV–visible absorption spectrum of purified 6-hydroxynicotine in 0.1-M HCl solution. **b** HPLC profile of purified 6-hydroxynicotine. **c** MS profile of purified 6-hydroxynicotine

The product 6-hydroxynicotine in the reaction mixture was extracted easily by dichloromethane after removing the cells in the reaction mixture by centrifugation and concentrating the supernatant by rotary evaporation under reduced pressure. Finally, 1.20 g of 6-hydroxynicotine was recovered from 600 mL of reaction mixture containing 2.6 g L^−1^ of 6-hydroxynicotine (76.9% recovery), where a total of approximately 1.43 g of nicotine was added during the reaction (overall yield, 83.9%, w/w). The purified product showed two characteristic maximum absorption peaks, at 232 and 295 nm, in 0.1 M HCl as indicated by UV–visible absorption spectrum (Fig. 8a), which corresponds to a previous report [34], as well as to authentic 6-hydroxynicotine (Fig. 2e). LC–MS determination showed a peak with *m/z* as 179.1198 ([M+H]^+^) that was identical to the calculated molecular mass of 6-hydroxynicotine (C_10_H_14_N_2_O, 178.1106; Fig. 8b, c). The purified product had an ^1^H-NMR spectrum (CDCl_3_, 600 MHz) as follows: *δ* 1.70 (m, 1H); 1.80 (m, 1H); 1.92 (m, 1H); 2.11 (m, 1H); 2.14 (s, 3H); 2.27 (q, *J* = 9.2 Hz, 1H); 2.85 (t, *J* = 8.3 Hz, 1H); 3.19 (td, *J* = 8.6, 1.8 Hz, 1H); 6.60 (d, *J* = 9.4 Hz, 1H); 7.27 (d, *J* = 2.4 Hz, 1H); 7.56 (dd, *J* = 9.4, 2.5 Hz, 1H); a ^13^C-NMR spectrum (CDCl_3_, 150 MHz): *δ* 22.3 (CH_2_), 33.7 (CH_2_), 40.0 (CH_3_), 56.6 (CH_2_), 67.6 (CH), 120.5 (C), 121.6 (CH), 132.7 (CH), 141.5 (CH), 165.3 (C) (Additional file 1: Figure S1). The NMR data were consistent with those from the authentic commercial standard 6-hydroxynicotine (Additional file 1: Figure S2) and previous study [9]. These results indicate that the purification of the product 6-hydroxynicotine from the reaction mixture can be achieved practically and easily with a satisfactory yield and purity.

## Conclusion

The disposal and detoxification of nicotine from tobacco and its wastes are a challenge for the tobacco industry. However, this biomass could be used as a potential resource to recover energy. In this study, we transformed nicotine from tobacco and its wastes into the valuable intermediate 6-hydroxynicotine using a nicotine-degrading *A. tumefaciens* S33 mutant, in which the enzyme NdhAB catalyzed nicotine hydroxylation with pseudoazurin as its electron acceptor. To accumulate quantities of the intermediate product, we identified the key enzyme Hno, responsible for further oxidative degradation of 6-hydroxynicotine, and disrupted its encoding gene to block the catabolism of 6-hydroxynicotine. With whole cells of the mutant as the biocatalyst, pseudoazurin, the electron acceptor for NdhAB, was regenerated by transferring its electrons to O_2_ via the respiration chain. At 30 °C and pH 7.0, nicotine was nearly completely transformed into 6-hydroxynicotine by the mutant whole cells; the biocatalyst with the highest nicotine transformation activity was obtained by growing the mutant in glucose–ammonium–HSP medium. The product was easily purified by dichloromethane extraction. In summary, we developed a novel green route for synthesizing the valuable chemical 6-hydroxynicotine from nicotine and providing an alternative strategy for utilizing tobacco and its wastes.

## Methods

### Bacterial strain, plasmids, and culture conditions

All bacterial strains, vectors, and recombinant plasmids in this study are listed in Table 2. *A. tumefaciens* S33, deposited at the China center for type culture collection (CCTCC) under accession number CCTCC AB 2016054 (originally CCTCC M 206131), was grown in “nicotine medium” or “nicotine–glucose–ammonium medium” (nicotine medium plus 1.0 g L^−1^ glucose, 0.2 g L^−1^ ammonium sulfate, and 1.0 g L^−1^ yeast extract) at 30 °C, as described previously [11, 35]. Nicotine was added to the media with a final concentration of 1.0 g L^−1^. 6-Hydroxynicotine medium and HSP medium contain 0.5 g L^−1^ 6-hydroxynicotine or HSP instead of nicotine in the nicotine medium, respectively, as the sole sources of carbon and nitrogen. *E. coli* cells were routinely grown in LB medium (10.0 g L^−1^ tryptone, 5.0 g L^−1^ yeast extract, and 10.0 g L^−1^ NaCl, pH 7.5) at 37 °C. Nicotine (> 99%) was obtained from Fluka (Buchs, Switzerland). HSP was purified from the culture broth of the nicotine-degrading *P. putida* S16 as described [12]. Authentic 6-hydroxynicotine was bought from Toronto Research Chemicals, Inc. (Toronto, Canada). All other chemicals were commercially available. The antibiotics and concentrations used were as follows: ampicillin (Ap), 100 mg L^−1^; gentamicin (Gm), 50 mg L^−1^.

**Table 2:** Strains and plasmids used in this study

Strain or plasmid	Description	Source
Strains		
*E. coli*		
BL21 (DE3)	F^−^ *ompT hsdS*(r_B_^−^ m_B_^−^) *gal dcm lacY1*(DE3)	Novagen
*A. tumefaciens*		
S33	Wild-type, nicotine degrader; G^−^	[4]
S33-∆*hno*	Gm^r^; *hno* mutant of strain S33	This study
S33-∆*hno*-C	Gm^r^; strain S33-∆*hno* containing pBBR-*hno*	This study
Plasmid		
pETDuet-1	Ap^r^; expression vector	Novagen
pETDuet-1-*hno*	Ap^r^; pETDuet-1 containing *hno* gene	This study
pJQ200SK	Gm^r^; mob^+^ *orip* 15A, lacZα^+^ sac B; suicide plasmid	[39]
pJQ-∆*hno*	Gm^r^; *hno* were disrupted and inserted into pJQ200SK	This study
pRK2013	Km^r^; helper plasmid for conjugation	Clontech
pBBR1MCS-5	Gm^r^; broad-host-range cloning vector	[40]
pBBR-*hno*	Gm^r^; *hno* inserted into pBBR1-MCS5	This study

### Enzyme assays

All assays were carried out in quartz cuvettes (1-cm light path) filled with 1 mL of reaction mixture at 30 °C using a UV–visible Ultrospec 2100 pro Spectrophotometer (GE Healthcare, USA). The reactions were initiated by the addition of enzyme. Ndh activity was determined as previously described [11] by monitoring the reduction of 2,6-dichlorophenolindophenolsodium (DCIP) with nicotine at 600 nm (*ε* = 21 mM^−1^ cm^−1^). The assay mixture contained 1 mM nicotine, 0.05 mM DCIP, and 50 mM phosphate buffer (pH 7.0). Hno activity was measured by detecting the formation of 6-hydroxypseudooxynicotine as previously reported [36]. The assay mixture contained 0.56 mM 6-hydroxynicotine, 100 mM NaCl, and 100 mM glycine–NaOH buffer (pH 9.2). The formation of 6-hydroxypseudooxynicotine was followed at 334 nm (*ε* = 20.7 mM^−1^ cm^−1^). One unit (U) of enzyme activity was defined as the amount of enzyme catalyzing the conversion 1 µmol of substrate per minute. Protein concentration was measured using the Bradford assay with bovine serum albumin as the standard [37].

### Purification of Hno from *A. tumefaciens* S33 and identification of its encoding gene

Cells were grown in nicotine–glucose–ammonium medium and disrupted by sonication according to the procedure for purifying NdhAB [24]. During the purification of NdhAB using DEAE Sepharose Fast Flow (GE Healthcare) [24], three yellow-colored fractions were eluted with 0.25 M NaCl concentration adjacent to the fractions containing NdhAB activity and were found to present Hno activity. The fractions with Hno activity were concentrated, desalted with 50 mM phosphate buffer (pH 7.0), and applied to a Q Sepharose column (GE Healthcare) equilibrated with 50 mM phosphate buffer (pH 7.0). The column was eluted at a 4-mL min^−1^ flow rate with five column volumes of the same buffer containing 0.1, 0.2, 0.25, 0.4, and 0.5 M NaCl as step gradients. Hno activity was eluted with NaCl of 0.25 M. After being concentrated and desalted, the enzyme was analyzed by SDS-PAGE. The purified enzyme was digested with trypsin and analyzed using MALDI-TOF MS analysis as described [24]. The results were searched against the genome of *A. tumefaciens* S33 [26] to identify the potential encoding genes of Hno.

### Heterologous expression and purification of Hno

The *hno* gene was amplified by PCR using the genomic DNA of strain S33 as the template. PCR primers (*hno*-F and *hno*-R, Table 3) were designed to incorporate restriction sites (*Bam*HI/*Hin*dIII) for subsequent ligation into an expression vector. The PCR products were then digested by *Bam*HI and *Hin*dIII and ligated into the expression vector pETDuet-1, which was then transformed into *E. coli* BL21 (DE3) for expression. The recombinant *E. coli* BL21 (DE3) strains were grown in LB at 37 °C to an optical density of 0.4–0.6 at 620 nm, and the induction was initiated by supplementing with 0.1 mM isopropyl-β-d-thiogalactopyranoside (IPTG) at 16 °C. After incubation for 12 h, cells were harvested and disrupted by sonication. The His-tagged protein was purified using a 5 mL HisTrap HP column (GE Healthcare). The target protein was eluted with a linear gradient of imidazole ranging from 50 to 200 mM in 20 mM NaH_2_PO_4_ (pH 7.0) buffer. To remove contaminants, active fractions were further applied to a DEAE Sepharose column that was washed with two column volumes of 50 mM phosphate buffer (pH 7.0) and then eluted with the same buffer containing 0.1, 0.2, 0.3, and 0.5 M NaCl by step gradients (two column volumes per each step). Hno activity was eluted with 0.2 M NaCl. The purity of the protein was detected by SDS-PAGE.

**Table 3:** Primers for expression, disruption, and complementation of the *hno* gene

Primers	Sequence (5′–3′)
*hno*-F	CGGGATCCGATGACAGAAAAGATATATGATGC (*Bam*HI)^a^
*hno*-R	CCCAAGCTTTTAAGCGGTCGCCTTC (*Hin*dIII)^a^
Primer A	CGCGGATCCATGACAGAAAAGATATATGATGC
Primer B	TCGATAAATGCGCGCTTTTAGGCTAGCTGCAACTCGTTC
Primer C	AAAAGCGCGCATTTATCGAACACGCCGAGATGGCTGAC
Primer D	GCCGGGCCCTTAAGCGGTCGCCTTC
Primer F2	GCCGGGCCCCATGACAGAAAAGATATATGATGC (*Apa*I)^a^
Primer R2	CGCGGATCCTTAAGCGGTCGCCTTC (*Bam*HI)^a^

^a^The restriction site is underlined

### Determination of the enzymatic reaction products

To identify the reaction products catalyzed by Hno, purified Hno was mixed with 11.2 mM 6-hydroxynicotine and 0.2 M NaCl in 1 mL of 50 mM phosphate buffer (pH 7.0) instead of glycine–NaOH because glycine produces an absorption peak in HPLC analysis, causing a negative effect on products separation. The reaction mixture was incubated at 37 °C for 30 min. The reaction was monitored by a UV–visible Ultrospec 2100 pro Spectrophotometer (GE Healthcare). The final products were identified by liquid chromatography-mass spectrometry (LC–MS) as described [24]. LC–MS data were obtained using a Finnigan Surveyor MSQ single quadrupole electrospray ionization mass spectrometer coupled with a Finnigan Surveyor HPLC (Finnigan/Thermo Electron Corporation, San Jose, CA, USA). Positively charged ions were detected. The HPLC system was equipped with an Agilent Eclipse XDB-C18 column (column size, 250 × 4.6 mm; particle size, 5 µM; Agilent, USA) and a PDA detector. A mixture of methanol and 12 mM formic acid (10:90, v/v) was used as the mobile phase, and the flow rate was set at 0.5 mL min^−1^.

### Deletion and complementation of the *hno* gene

The deletion of the *hno* gene was performed using the in-frame deletion system based on homologous recombination [24]. First, a 503-bp upstream sequence (primers A and B in Table 3) and a 485-bp downstream sequence (primers C and D in Table 3) were amplified by PCR using the genome of *A. tumefaciens* S33 as template. The PCR products above were mixed together for another seven amplifying cycles to obtain truncated *hno* genes due to the 19-bp complementary sequences between primers B and C. The truncated *hno* genes were used as a template for the third PCR to procure more of the shortened target genes and were then inserted into the suicide plasmid pJQ200SK with restriction sites *Bam*HI and *Apa*I, generating the recombinant plasmid pJQ-∆*hno*. The recombinant plasmid pJQ-∆*hno* was transferred into *A. tumefaciens* S33 by conjugation with the help of *E. coli* HB101(pRK2013). The mutant with single-crossover DNA exchange was obtained by the Gm-resistance screening. After culturing the mutant in HSP medium containing 20% sucrose (w/v), double-crossover DNA exchange was achieved, and the plasmid pJQ200SK with completed *hno* gene was removed, generating *A. tumefaciens* S33-∆*hno* which is sensitive to Gm.

To recover the activity of Hno for *A. tumefaciens* S33-∆*hno*, the complete *hno* genes were amplified from the genome of *A. tumefaciens* S33 using the primers F2 and R2 (see Table 3). The PCR products were digested by *Apa*I and *Bam*HI and inserted into the plasmid of pBBR1MCS-5 with the same restriction sites to procure the complementation plasmid pBBR-*hno*. Then the recombinant plasmid was transferred into *A. tumefaciens* S33-∆*hno* through electroporation transformation (100 μL competent cells, 1 μg plasmid DNA, 1.2 kV cm^−1^ field strength, 200 Ω resistance, 25 μF capacitance, Bio-Rad Gene Pulser Xcell™ system, Hercules, CA, USA) [38], obtaining the complementation strain *A. tumefaciens* S33-∆*hno*-C.

To further confirm the deletion of the *hno* gene, the wild-type strain *A. tumefaciens* S33, and the engineered strains, *A. tumefaciens* S33-∆*hno* and *A. tumefaciens* S33-∆*hno*-C, were cultured in LB, HSP, nicotine, and 6-hydroxynicotine media. For LB, HSP, and nicotine media, optical density (OD) at 620 nm was measured. For 6-hydroxynicotine medium, a solid agar plate was used to observe cell growth. Then the three strains were cultured in HSP medium or nicotine–glucose–ammonium medium. Cells were collected by centrifugation at 30,000×*g* for 10 min and disrupted by sonication. The Ndh and Hno activities were determined, as described above.

### Extraction of crude nicotine from tobacco waste

The nicotine used as the substrate for the biotransformation reactions in this study was prepared from tobacco waste according to a previous report [12]. The tobacco waste containing 2.3% (w/w) nicotine was obtained from the Yuxi Cigarrette Co. Ltd., Yunnan Province, China. Nicotine was separated by steam distillation, purified by extraction with chloroform, and then evaporation was used to recover the solvent. The final product contained 94–97% nicotine.

### Test of the reaction catalyzed by the whole cells of *A. tumefaciens* S33-∆*hno*

To test the possibility of biotransformation of nicotine into 6-hydroxynicotine, the whole cells (~ 1.45 g L^−1^ DCW) of *A. tumefaciens* S33-∆*hno* were used as a catalyst. These cells were prepared by growing in nicotine–glucose–ammonium medium for 24 h. The reaction was performed at 30 °C in 50 mM sodium phosphate buffer (pH 7.0) with 0.85 g L^−1^ nicotine as the substrate. Concentrations of nicotine and the expected product 6-hydroxynicotine were determined by HPLC (Agilent 1100 system, equipped with an Eclipse XDB-C18 column and a PDA detector). The mobile phase included methanol and 8 mM formic acid (10:90, v/v) with a flow rate of 0.5 mL min^−1^ at 30 °C.

### Optimization of the reaction conditions for the biotransformation of nicotine into 6-hydroxynicotine by whole cells of *A. tumefaciens* S33-∆*hno*

To improve the catalytic rate to produce 6-hydroxynicotine, different reaction conditions were examined. Routinely, the reactions were carried out at 30 °C in 50 mM sodium phosphate buffer (pH 7.0) with 1.0 g L^−1^ nicotine as the substrate and ~ 1.5 g L^−1^ DCW whole cells of *A. tumefaciens* S33-∆*hno* as a catalyst, which were grown in nicotine–glucose–ammonium medium for 24 h. The examined reaction conditions were as follows: The amount of *A. tumefaciens* S33-∆*hno* whole cells used for the catalyst was tested in a range from 0.3 to 4.8 g L^−1^ DCW. The tested initial concentration of nicotine increased from 1 to 16 g L^−1^. The buffers used were changed from pH 6.0 to 10.0, where 50 mM sodium phosphate buffer (pH 6.0 and pH 7.0), 50 mM Tris–HCl buffer (pH 8.0), and 50 mM glycine–NaOH buffer (pH 9.0 and pH 10.0) were prepared. The catalytic temperature varied from 16 °C to 44 °C. The oxygen requirement was tested in a rotary shaker with the rotation speed set at 0, 100, 200, or 250 rpm. Moreover, to obtain the biocatalyst with the highest activity, various media were tested for growing *A. tumefaciens* S33-∆*hno* including HSP medium, glucose–ammonium medium, LB medium, and all these media plus 0.1 g L^−1^ of HSP or nicotine. The amounts of the substrate and product in the reaction mixtures were measured by HPLC as above.

### Batch and fed-batch biotransformation

To re-utilize the biocatalyst, batch and fed-batch biotransformation were tried. Cells of *A. tumefaciens* S33-∆hno (~ 1.0 g L^−1^ DCW), cultured with glucose–ammonium medium plus 0.1 g L^−1^ HSP, were added as a catalyst to 200 mL of 50 mM sodium phosphate buffer (pH 7.0) containing 1.0 g L^−1^ nicotine. The reaction was performed at 30 °C with a speed of 200 rpm. For the batch biotransformation, the biocatalyst was recycled by centrifugation to collect the cells and then was re-used three times. For the fed-batch biotransformation, nicotine was supplemented three times at a concentration of 1.0 g L^−1^ after the substrate was almost completely consumed.

### Extraction and purification of the product 6-hydroxynicotine

After the reaction finished, the supernatant was obtained by centrifugation at 10,000×*g* for 10 min and adjusted to pH 10.5 with 2 M NaOH. Then the supernatant was concentrated by rotary evaporation under reduced pressure at 60 °C. The product 6-hydroxynicotine was extracted from the concentrate with equal volume of dichloromethane for three times. The extracts were pooled, and then was dried using Na_2_SO_4_. The solvent was removed further by evaporation. The isolated product was identified by recording the UV–visible absorption spectra after being dissolved in a 0.1-M HCl solution and determining its mass spectrometry by LC–MS on the Dionex’s UltiMate 3000 UHPLC-Bruker’s impact HD high-resolution mass spectrometry system. The same column and chromatography conditions used above were applied. The NMR spectra were recorded for solutions in CDCl_3_ on an AVANCE 600 spectrometer (Bruker, Switzerland) operating at 600 MHz for ^1^H and at 150 MHz for ^13^C.

### Nucleotide sequence accession numbers

The *A. tumefaciens* S33 genome GenBank accession numbers are CP014259.1 and CP014260.1.

## Additional file

**Additional file 1: Figure S1.** NMR spectra of the product 6-hydroxynicotine. **a** ^1^H-NMR spectrum (CDCl_3_, 600 MHz). **b** ^13^C-NMR spectrum (CDCl_3_, 150 MHz). **Figure S2.** NMR spectra of the authentic commercial standard 6-hydroxynicotine. **a** ^1^H-NMR spectrum (CDCl_3_, 600 MHz). **b** ^13^C-NMR spectrum (CDCl_3_, 150 MHz).

## Abbreviations

DCIP: 2,6-dichlorophenolindophenolsodium
DCW: dry cell weight
FPKM: fragments per kilobase per million
Hno: 6-hydroxynicotine oxidase
HSP: 6-hydroxy-3-succinoylpyridine
IPTG: isopropyl-β-d-thiogalactopyranoside
NdhAB: nicotine dehydrogenase
OD: optical density
PDA: photodiode array
rpm: rev min^−1^
