# Supplementary material for: Green route to synthesis of valuable chemical 6-hydroxynicotine from nicotine in tobacco wastes using genetically engineered Agrobacterium tumefaciens S33
Source: Biotechnol Biofuels. 2017 Dec 4;10:288. doi: 10.1186/s13068-017-0976-9 (PMC5713474; doi:10.1186/s13068-017-0976-9)
Supplement: Supplementary file 1 — Additional file 1: Figure S1. NMR spectra of the product 6-hydroxynicotine. a 1H-NMR spectrum (CDCl3, 600 MHz). b 13C-NMR spectrum (CDCl3, 150 MHz). Figure S2. NMR spectra of the authentic commercial standard 6-hydroxynicotine. a 1H-NMR spectrum (CDCl3, 600 MHz). b 13C-NMR spectrum (CDCl3, 150 MHz). [file 13068_2017_976_MOESM1_ESM.docx]

**Additional file 1**

**Green route to synthesis of valuable chemical 6-hydroxynicotine from nicotine in tobacco wastes using genetically engineered *Agrobacterium tumefaciens* S33**

Wenjun Yu ^1†^, Rongshui Wang ^1†^, Huili Li ^1^, Jiyu Liang ^1^, Yuanyuan Wang ^2^, Haiyan Huang ^2^, Huijun Xie ^3^ and Shuning Wang ^1,^*

^1^State Key Laboratory of Microbial Technology, School of life science, Shandong University, Jinan 250100, People’s Republic of China, ^2^Institute of Basic Medicine, Shandong Academy of Medical Science, Jinan 250062, People’s Republic of China, ^3^Environment Research Institute, Shandong University, Jinan 250100, People’s Republic of China

†Wenjun Yu and Rongshui Wang contributed equally to this work

* Correspondence: shuningwang@sdu.edu.cn

**a**


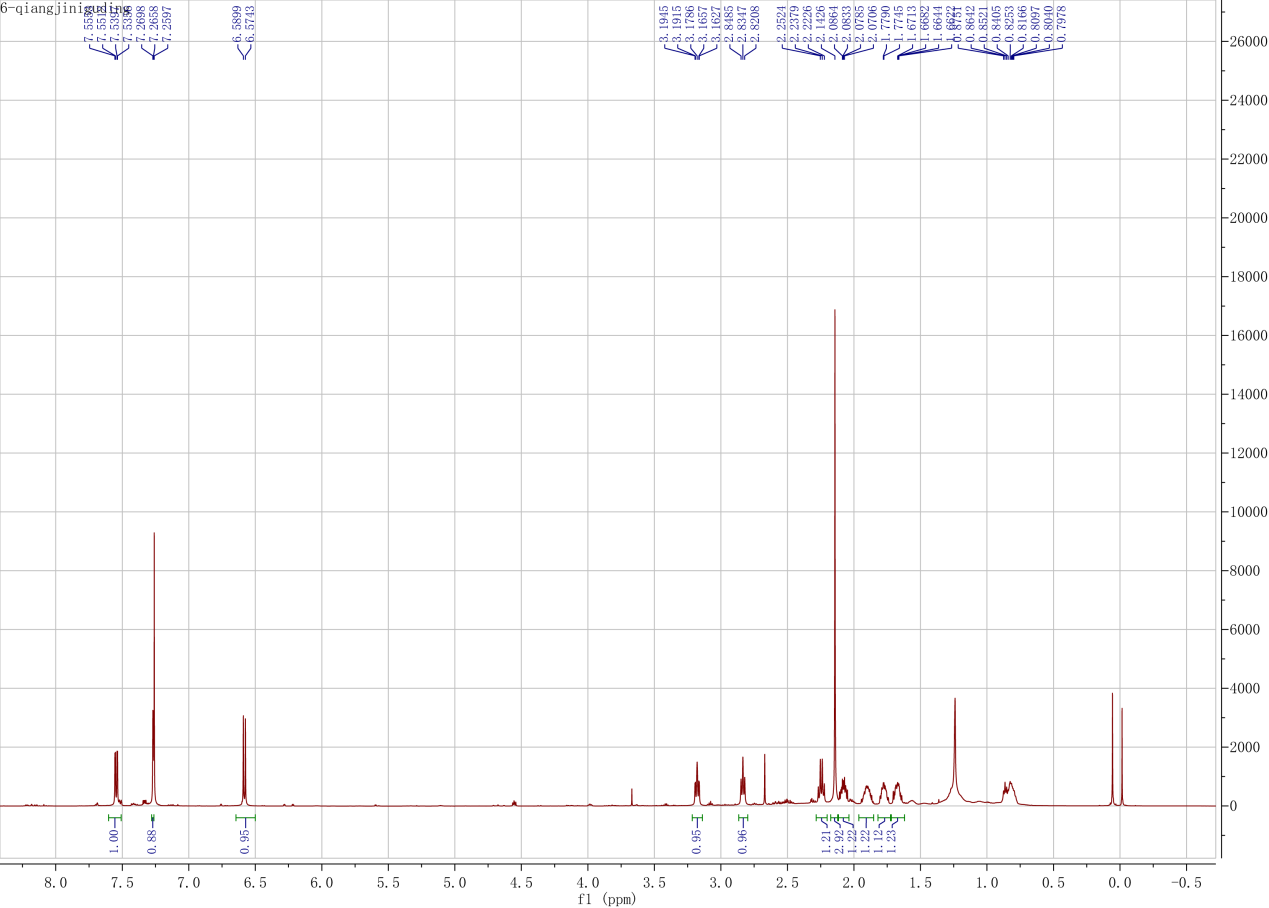


**b**


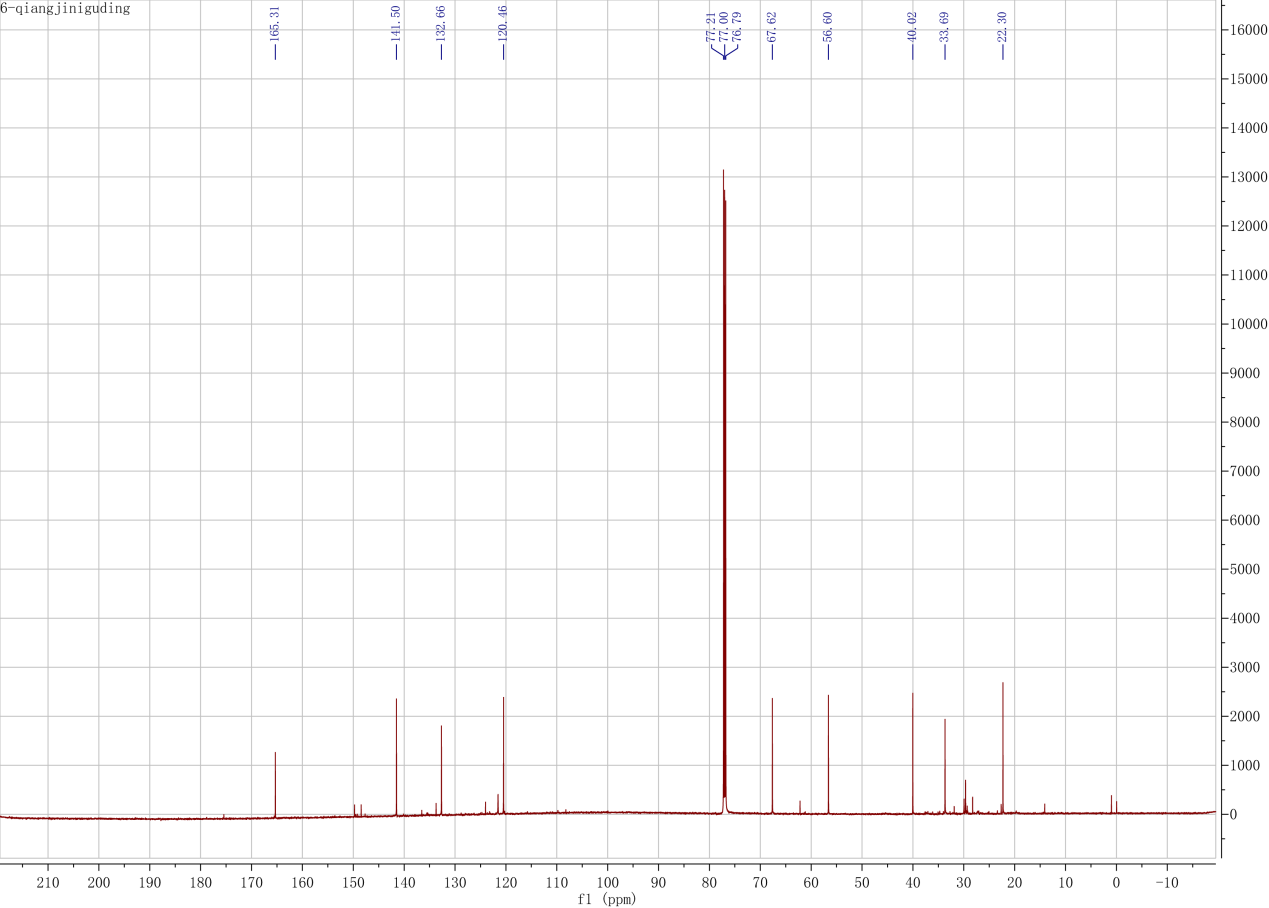


**Figure S1** NMR spectra of the product 6-hydroxynicotine prepared in this study. **a** ^1^H-NMR spectrum (CDCl_3_, 600 MHz). **b** ^13^C-NMR spectrum (CDCl_3_, 150 MHz)

**a**


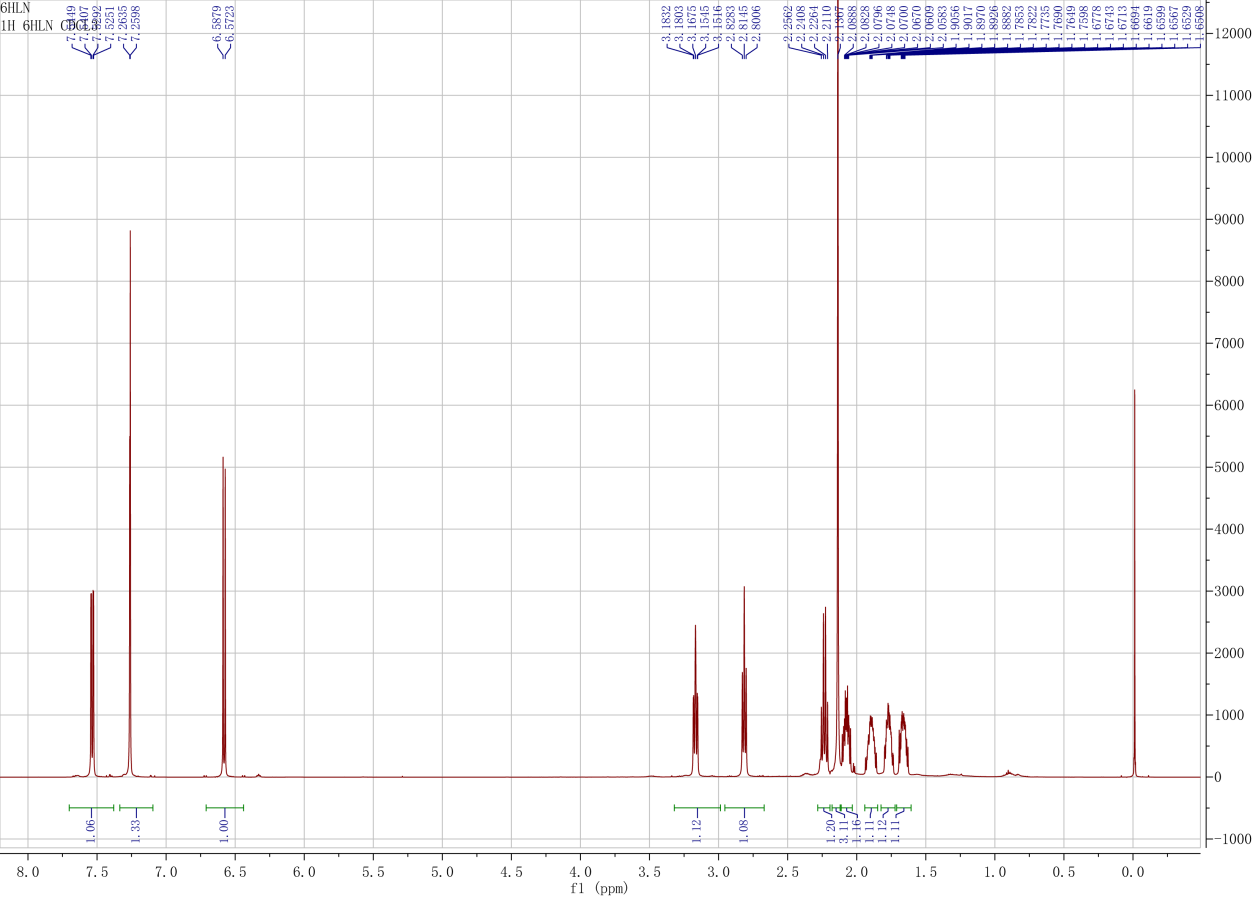


**b**


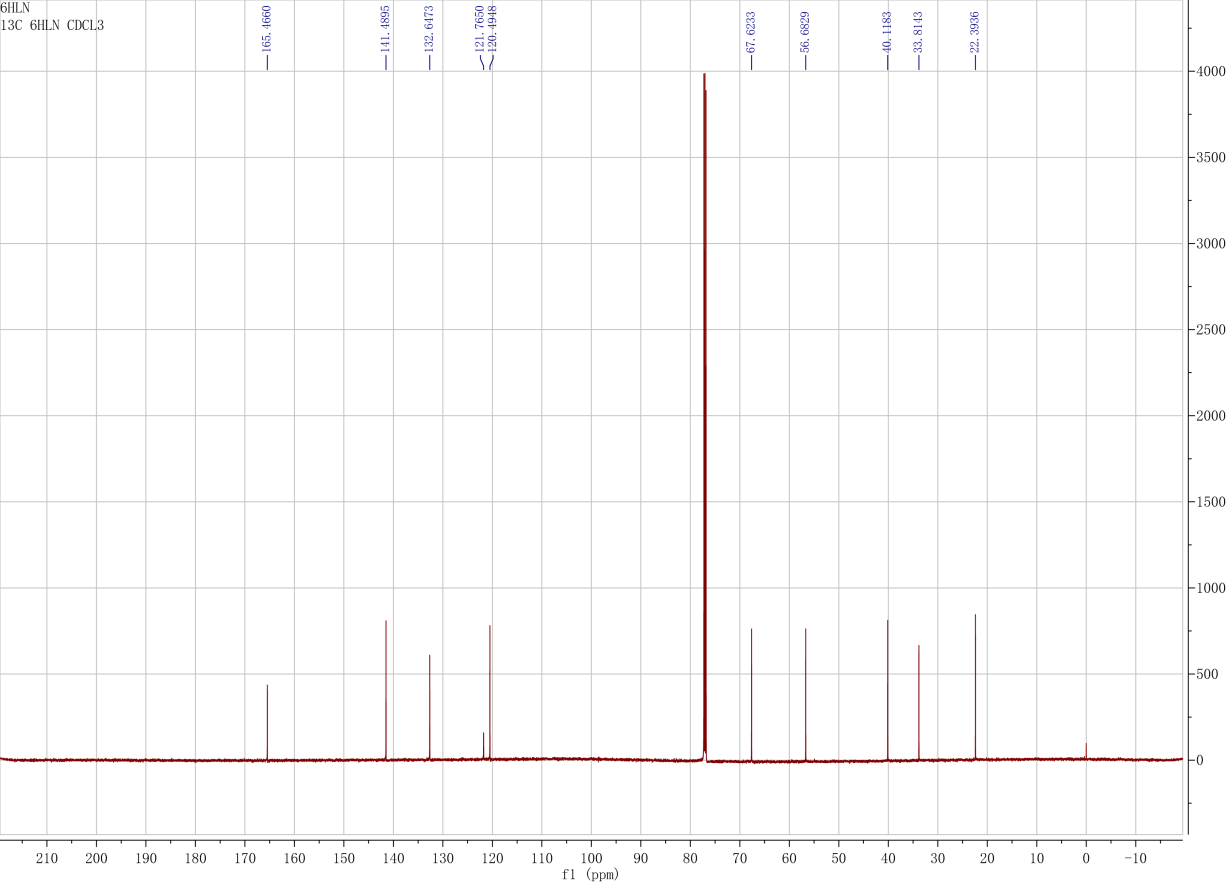


**Figure S2** NMR spectra of the authentic commercial standard 6-hydroxynicotine. **a** ^1^H-NMR spectrum (CDCl_3_, 600 MHz). **b** ^13^C-NMR spectrum (CDCl_3_, 150 MHz). ^1^H NMR (600 MHz, CDCl_3_) 1.69 (m, 1H), 1.80 (m, 1H), 1.94 (m, 1H), 2.10 (m, 1H), 2.14 (s, 3H), 2.23 (q, *J* = 9.2 Hz, 1H), 2.83 (t, *J* = 8.3 Hz, 1H), 3.18 (td, *J* = 8.6, 1.7 Hz, 1H), 6.59 (d, *J* = 9.4 Hz, 1H), 7.26 (d, *J* = 2.2 Hz, 1H), 7.54 (dd, *J* = 9.4, 2.5 Hz, 1H). ^13^C NMR (150 MHz, CDCl_3_) δ 22.4 (CH_2_), 33.8 (CH_2_), 40.1 (CH_3_), 56.7 (CH_2_), 67.6 (CH), 120.5 (C), 121.8 (CH), 132.7 (CH), 141.5 (CH_2_), 165.5 (C)
